# Harnessing the Power of Microbiota: How Do Key *Lactobacillus* Species Aid in Clearing High-Risk Human Papilloma Virus Infection and Promoting the Regression of Cervical Dysplasia?

**DOI:** 10.3390/biology14081081

**Published:** 2025-08-19

**Authors:** Edyta Kęczkowska, Joanna Wrotyńska-Barczyńska, Aneta Bałabas, Magdalena Piątkowska, Michalina Dąbrowska, Paweł Czarnowski, Ewa E. Hennig, Maciej Brązert, Piotr Olcha, Michał Ciebiera, Natalia Zeber-Lubecka

**Affiliations:** 1 Warsaw Institute of Women’s Health, 00-189 Warsaw, Poland; 2 Department of Diagnostics and Treatment of Infertility, Poznan University of Medical Sciences, 60-535 Poznan, Poland; 3 Department of Genetics, Maria Sklodowska-Curie National Research Institute of Oncology, 02-781 Warsaw, Poland; 4 Department of Gastroenterology, Hepatology and Clinical Oncology, Center of Postgraduate Medical Education, 02-781 Warsaw, Poland; 5 Department of Gynaecology and Gynaecological Endocrinology, Medical University of Lublin, 20-049 Lublin, Poland; 6 Second Department of Obstetrics and Gynecology, Center of Postgraduate Medical Education, 00-189 Warsaw, Poland

**Keywords:** HPV infection, cervical microbiota, cervical dysplasia, *Lactobacillus*

## Abstract

*Lactobacillus* species play a fundamental role in maintaining a healthy vaginal microbiota and have been increasingly recognized for their protective effects against high-risk human papillomavirus (HR-HPV) infection and the progression of cervical intraepithelial neoplasia (CIN). These beneficial bacteria contribute to host defense through multiple mechanisms, including the production of lactic acid that sustains a low vaginal pH, enhancement of epithelial barrier integrity via E-cadherin regulation, and modulation of immune signaling pathways such as interferon responses and NF-κB activity. *Lactobacillus* strains exert anti-inflammatory effects by downregulating pro-inflammatory cytokines and interfering with oncogenic pathways including Wnt/β-catenin and the expression of HPV E6 and E7 proteins. Additionally, they may regulate tumor-suppressor microRNAs and modulate dendritic cell and macrophage activity, supporting antiviral immunity. Recent studies have explored their potential influence on CIN regression and HR-HPV clearance, particularly the strains *Lactobacillus crispatus* and *L. gasseri*, which are associated with favorable microbial community states. This review explores the potential mechanisms through which *Lactobacillus* species contribute to HR-HPV clearance and the regression of cervical dysplasia, integrating evidence from molecular studies, in vivo models, and clinical trials. The emerging role of probiotic interventions as adjunctive strategies in HPV management is also discussed, highlighting their possible synergy with conventional treatments and prophylactic vaccination.

## Simple Summary

This review explores the potential mechanisms through which *Lactobacillus* species contribute to high-risk human papillomavirus clearance and the regression of cervical dysplasia. We discuss their role in modulating local immune responses, producing antiviral metabolites, and restoring microbiota balance—factors that collectively enhance the host’s ability to combat infection and reverse precancerous lesions.

## 1. Introduction

Cervical cancer remains a significant global health burden, ranking among the leading causes of cancer-related death in women, particularly in low- and middle-income countries. A persistent infection with high-risk human papillomavirus (HR-HPV), most notably types 16 and 18, is a central etiological factor in the development of cervical intraepithelial neoplasia (CIN) and cervical cancer [1]. While prophylactic HPV vaccines have substantially reduced infection rates in vaccinated populations, there is a critical lack of effective therapeutic strategies for those already infected [2]. Although most HR-HPV infections resolve spontaneously, a subset of them persist and progress, underscoring the need for novel, non-invasive interventions that can promote viral clearance and prevent a malignant transformation [3].

In recent years, mounting evidence has revealed the pivotal role of the cervicovaginal microbiota in modulating susceptibility to an HR-HPV infection, persistence, and the progression of cervical dysplasia [4]. A microbiota dominated by *Lactobacillus* species—particularly *Lactobacillus crispatus*, *L. gasseri*, *L. jensenii*, and, to a more complex degree, *L. iners*—is characterized by a protective mucosal environment that fosters immune regulation, epithelial integrity, and pathogen resistance [5]. Conversely, numerous studies showed that a vaginal microbiota dominated by *Lactobacillus iners* or non-lactobacilli was linked to a 3–5 times higher risk of an HPV infection, a 2–3 times higher risk of an HR-HPV infection, and an increased likelihood of CIN progression and carcinogenesis compared to a microbiota dominated by *L. crispatus* [6]. In contrast, microbial dysbiosis, often characterized by increased microbial diversity and the depletion of *Lactobacillus*, was correlated with HR-HPV persistence and a greater risk of high-grade lesions [7]. However, the relationship between the microbiota and HR-HPV is far from uniform. Substantial differences in microbiota composition occur across geographic, ethnic, and sociodemographic lines [8]. For example, non-*Lactobacillus*-dominant profiles—more frequently observed in women of African descent—were linked with a higher HR-HPV burden and might necessitate tailored therapeutic approaches [9]. These disparities challenge the one-size-fits-all model of microbiota modulation and call for a more nuanced, equity-informed approach.

Concurrently, the field is witnessing a paradigm shift from generic probiotic supplementation to precision microbiota strategies that leverage host–microbiota–virus interactions. Advances in omics technologies, such as metagenomics and metabolomics, are uncovering functional insights that could enable individualized interventions based on microbial biomarkers, host immune status, and ecological context [10]. Such translational developments offer a promising frontier for non-invasive, microbiota-centered therapeutics.

## 2. HR-HPV Infection and Cervical Dysplasia: Virological and Oncogenic Foundations

HPVs are small, non-enveloped viruses, approximately 55 nm in diameter, belonging to the Papillomaviridae family. Their genome consists of circular, double-stranded DNA enclosed within an icosahedral capsid [11]. HPVs are classified into five genera (i.e., Alpha-, Beta-, Gamma-, Mu-, and Nupapillomaviruses) based on genomic features and tropism. The Mu and Nu types typically cause benign cutaneous lesions, while Beta and Gamma HPVs infect the skin [12]. As regards the Alpha types, some are mucosotropic and are strongly associated with oncogenesis. To date, more than 230 HPV genotypes have been described and are broadly categorized into low-risk (LR)-HPV and HR-HPV types, depending on their oncogenic potential [13]. A persistent infection with HR-HPV genotypes is the principal etiological factor in the development of CIN, a spectrum of premalignant lesions in the cervical epithelium. If unresolved, such dysplastic lesions may progress to invasive cervical cancer, although the process may span many years [14]. Seventeen genotypes are implicated in the precancerous lesions, with HPV 16 and 18 being the most prevalent, followed by types such as HPV 31, 33, 45, 52, and 58, as well as a group of less common HR types [15]. LR-HPV types, notably 6 and 11, are typically associated with benign lesions such as genital warts [15].

The HPV genome, approximately 7000 to 8000 base pairs in length, comprises three functional regions, as follows: one encoding early proteins (E1–E7) involved in viral replication and host cell manipulation; another one encoding structural capsid proteins (L1 and L2); and a regulatory long control region responsible for the initiation of transcription and replication [11]. Productive HPV infection refers to the stage at which the virus actively replicates and produces viral particles, typically occurring in differentiated epithelial cells [16]. In contrast, non-productive infection is characterized by the presence of viral DNA without active replication or virion production, often associated with viral persistence and potential progression to neoplasia [17]. An HPV infection begins when viral particles gain access to the basal epithelial layer via microabrasions and bind to cell surface receptors, including heparan sulfate proteoglycans and integrins, facilitating endocytic uptake [18]. Once inside the host cell, the viral genome is delivered to the nucleus, where early genes are expressed to establish and maintain infection in proliferating basal cells. As infected cells migrate and differentiate, viral replication intensifies and late gene expression ensues, leading to virion assembly and eventual release through epithelial desquamation [19].

**Figure 1:**
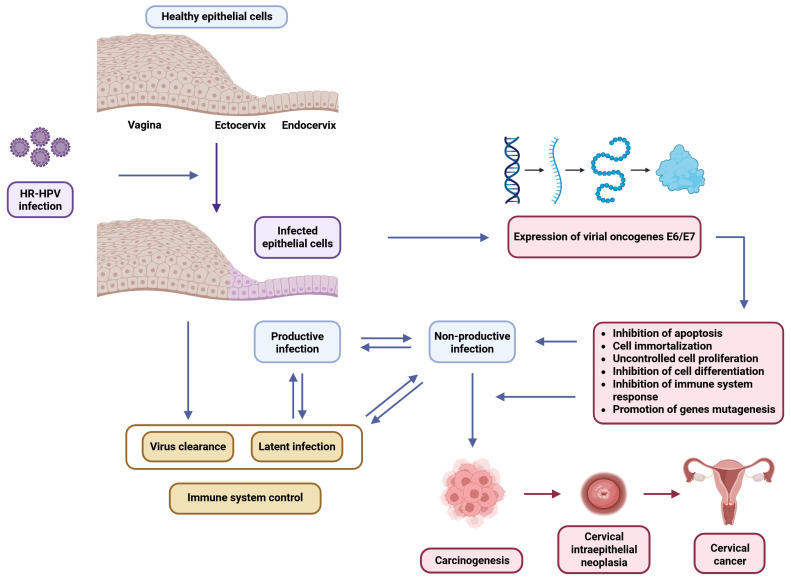
Schematic representation of the progression of a high-risk (HR)-HPV infection in epithelial cells. Following the initial infection, the virus may cause a subclinical infection leading to either productive or non-productive lesions. Outcomes depend on immune control and may include viral clearance, a latent infection, or progression to carcinogenesis. The dysregulation of E6/E7 oncogene expression plays a key role by promoting cell immortalization, abnormal proliferation, the inhibition of apoptosis and differentiation, immune evasion, and gene mutations.

The oncogenic potential of HR-HPV lies in the expression of the following three key early proteins: E6, E7, and E5 [20]. These oncoproteins are often overexpressed following the integration of viral DNA into the host genome, a process that disrupts the normal viral life cycle and typically results in the deletion of regulatory regions such as E1 and E2, while preserving E6 and E7 coding sequences [21]. E6 promotes the degradation of p53, thereby impairing cell cycle arrest and apoptosis and, additionally, modulates immune evasion, autophagy, and cellular metabolism. E7 inactivates the retinoblastoma protein (pRb), enabling uncontrolled cell cycle progression and enhancing cellular proliferation [22]. It also affects immune recognition and increases cellular migration. E5 contributes to oncogenesis by enhancing epidermal growth factor receptor (EGFR) signaling, promoting angiogenesis through vascular endothelial growth factor (VEGF) upregulation, and suppressing immune responses [20]. Together, these proteins manipulate host cell pathways to promote immune evasion, sustained proliferation, resistance to apoptosis, and, ultimately, cellular transformation (Figure 1).

Cytological hallmarks of an HPV infection include the presence of koilocytes, i.e., squamous epithelial cells with enlarged, hyperchromatic nuclei surrounded by a perinuclear halo [23]. In dysplastic cells, morphological features become more pronounced, including cellular pleomorphism, an increased nuclear-to-cytoplasmic ratio, irregular nuclear contours, thin nuclear membranes, nuclear indentations and vacuolization, prominent nucleoli, abnormal mitotic figures, disorganized epithelial stratification, the loss of polarity, and an increased mitotic index [24,25]. In line with WHO and ASCCP criteria, CIN is categorized by epithelial involvement depth, as follows: CIN1 (low-grade squamous intraepithelial lesion, LSIL) presents as mild dysplasia affecting only the basal third of the squamous epithelium and is typically transient; CIN2 (high-grade squamous intraepithelial lesion, HSIL) represents moderate dysplasia involving up to two-thirds of the epithelial thickness, with reduced chances of spontaneous regression; and CIN3 (HSIL) constitutes severe dysplasia or carcinoma in situ, where atypical cells span the entire epithelial layer without breaching the basement membrane [26].

While CIN1 and a significant proportion of CIN2 lesions may regress within 24 months, particularly in HR-HPV-negative women under 30, the regression is influenced by several protective factors [27]. These include nonsmoking status, nulliparity, and a vaginal microbiota dominated by *Lactobacillus* spp. A meta-analysis from 2021 revealed regression rates of 60% for CIN1, 55% for CIN2, and 28% for CIN3. The corresponding progression rates to higher-grade lesions were 11%, 19%, and 2%, respectively, with invasive cancer developing in <0.5% of CIN1/2 cases [28]. Progression is more likely when an HR-HPV infection, especially with type 16, coexists with immunosuppression (an HIV infection and immunosuppressive therapy), behavioral and environmental exposures (smoking and long-term oral contraceptive use), or certain host genetic factors. For instance, a co-infection with *Chlamydia trachomatis*, *Mycoplasma hominis*, *Trichomonas vaginalis*, and herpesviruses (HSV and EBV) may act as a cofactor by impairing HPV clearance or promoting dysplastic transformation [29,30,31,32,33]. Although EBV does not share the same primary infection site as HPV, it establishes lifelong latent infection and modulates host immune responses. EBV oncogenic proteins, including latent membrane protein 1 (LMP1), activate multiple signaling pathways and epigenetic mechanisms that contribute to immune evasion and chronic inflammation, indirectly facilitating HPV persistence and neoplastic progression [34,35].

## 3. Vaginal Microbiota: Community State Types and Dysbiotic Conditions

The vaginal environment constitutes a unique microecosystem predominantly colonized by Gram-positive bacteria [36]. *Lactobacillus* species, members of the *Lactobacillaceae* family, are the predominant constituents of this microbiota and play a crucial role in maintaining vaginal health [37]. The key species include *L. crispatus*, *L. iners*, *L. jensenii*, and *L. gasseri*, which act as natural probiotics employing both direct and indirect mechanisms that inhibit the overgrowth of pathogens and preserve microbial balance [37]. These bacteria produce lactic acid, which lowers the vaginal pH to below 4.5, creating an acidic environment that is inhospitable to many pathogenic microorganisms. Certain *Lactobacillus* strains also synthesize hydrogen peroxide (H_2_O_2_), a potent antimicrobial compound that further contributes to microbial homeostasis [38]. Additionally, *Lactobacillus* spp. secrete bacteriocins (i.e., antimicrobial peptides that inhibit the growth of competing bacteria), thereby reinforcing the dominance of beneficial microbes [39]. Another important mechanism involves the adherence of *Lactobacillus* to the vaginal epithelial cells, forming a protective biofilm that prevents colonization by pathogenic species [40]. Moreover, these bacteria modulate local immune responses by promoting the production of host-derived antimicrobial peptides and influencing immune cell activity, thereby supporting immune equilibrium [41]. Disruptions to these protective mechanisms, caused by factors such as menstruation, pregnancy, sexual activity, antibiotic use, or vaginal douching, may disturb the microbial balance and predispose individuals to conditions such as BV, sexually transmitted infections, and vulvovaginal candidiasis [42,43,44]. Thus, preserving the functional integrity of *Lactobacillus* species is essential for maintaining vaginal health. In clinical settings, BV is primarily diagnosed using the Amsel criteria, which involve assessing the following four key factors: vaginal pH, vaginal discharge characteristics, the presence of clue cells (vaginal epithelial cells covered with bacteria), and the result of the “whiff test” (a fishy odor detected when 10% potassium hydroxide is added to a vaginal discharge sample) [45,46]. Among these, vaginal pH > 4.5 and the presence of a thin, homogeneous, and milky discharge are the most sensitive indicators (97%), though the latter has a low specificity (26%) and a positive predictive value (27%) [46,47]. The presence of clue cells is the most specific criterion (86%). When at least three of these criteria are met, BV diagnosis becomes more reliable, with a sensitivity of 97% and specificity of 90% [46,47]. BV was associated with an increased risk of sexually transmitted infections caused by pathogens such as *Neisseria gonorrhoeae*, *Chlamydia trachomatis*, *Trichomonas vaginalis*, HPV, herpes simplex virus, and human immunodeficiency virus [48]. Aerobic vaginitis (AV) is another form of vaginal dysbiosis. It also disrupts the normal *Lactobacillus*-dominated microbiota but presents with distinct characteristics [49]. Unlike BV, AV is marked by an overt inflammatory response, infiltration of leukocytes and parabasal cells, and the overgrowth of aerobic bacteria, such as *Escherichia coli*, *Enterococcus*, *Staphylococcus aureus*, and group B *Streptococcus* [50]. AV was described as the aerobic counterpart of BV, as it also involves reduced lactic acid levels due to *Lactobacillus* depletion [47].

Advances in molecular techniques, particularly next-generation sequencing, have enabled the identification of previously uncultivable and fastidious bacterial species, leading to the classification of distinct vaginal microbial community state types (CSTs) [51,52,53]. In reproductive-aged women, these CSTs are typically categorized into five major groups based on the composition and relative abundance of vaginal bacterial species. CST I, CST II, CST III, and CST V are defined by the dominance of *Lactobacillus crispatus*, *L. gasseri*, *L. iners*, and *L. jensenii*, respectively. In contrast, CST IV is characterized by a diverse community of facultative anaerobes and a reduced presence of *Lactobacillus* spp. [51]. CST IV is further subdivided into the following two subtypes: CST IV-A, comprising genera such as *Anaerococcus*, *Peptoniphilus*, *Corynebacterium*, *Prevotella*, *Finegoldia*, and *Streptococcus*, and CST IV-B, which includes taxa such as *Atopobium*, *Gardnerella*, *Sneathia*, *Mobiluncus*, *Megasphaera*, and other members of the order Clostridiales [54]. According to the Nugent scoring system, CST IV is commonly associated with bacterial vaginosis (BV), a dysbiotic condition [49]. However, this CST was also observed in some asymptomatic individuals, particularly among Black and Hispanic women, where it accounted for approximately 40% of cases [55]. This observation raises a question whether CST IV represents a non-pathogenic variation in the vaginal microbiota or an asymptomatic form of BV, underscoring the need to refine current diagnostic and classification criteria.

Building on the earlier discussion of BV and CSTs, growing evidence suggests that vaginal dysbiosis may also contribute to the development of cervical cancer [56,57,58]. A study investigating cervicovaginal metabolomic profiles revealed three distinct clusters associated with inflammation-driven, dysbiotic cervical carcinogenesis [59]. These metabolic signatures were correlated with vaginal pH and an HPV infection status. Elevated concentrations of 3-hydroxybutyrate, eicosenoate, and oleate/vaccenate were linked to disease progression, whereas increased levels of sphingolipids, plasmalogens, and linoleate reflected an inflammatory state [59]. A microbiota dominated by non-*Lactobacillus* species was associated with altered amino acid and nucleotide metabolism, promoting a microenvironment favorable to HPV persistence and inflammation. Conversely, *Lactobacillus*-dominant communities were linked to higher levels of adenosine and cytosine and lower levels of inflammatory markers, indicating a more protective profile. Additionally, the presence of specific metabolites such as glycochenodeoxycholate and carnitine in the non-*Lactobacillus*-dominated microbiota appeared to exacerbate inflammation, further contributing to carcinogenic processes. This area of research continues to evolve, offering new insights into how microbial communities might influence disease progression [59].

## 4. The Role of *Lactobacillus* in HR-HPV Clearance and CIN Regression

*Lactobacillus* species are central to the maintenance of a healthy vaginal microbiota and have been increasingly recognized for their role in natural defense against persistent HR-HPV infection and the progression of CIN. One of the primary mechanisms by which *Lactobacillus* exerts its protective effect is through the production of lactic acid, which maintains a low vaginal pH [60]. This acidic environment is hostile to many pathogens and prevents the overgrowth of anaerobic bacteria that are associated with dysbiosis and chronic inflammation [61]. The mechanisms by which *Lactobacillus* species protect against an HPV infection, persistence, and progression to cervical cancer are multifaceted and involve several pathways. *Lactobacillus* species promote the integrity of the epithelial mucosal barrier by increasing E-cadherin levels, a biomarker of epithelial health [62,63]. This reduces the risk of HPV’s penetration through abrasions or epithelial disruptions. *Lactobacillus* enhances the antiviral innate immunity by upregulating interferon (IFN)-α and IFN-β expression through pathways like NF-κB [64,65]. These bacteria decrease the levels of pro-inflammatory cytokines such as interleukin (IL)-6 and IL-1β, reducing chronic inflammation and the associated HPV persistence risks [66]. Butyrate production by *Lactobacillus* species helps restore immunity via interferon regulatory factor 3 (IRF3)-dependent pathways, aiding HPV clearance [60]. Certain *Lactobacillus* species (*L. crispatus* and *L. gasseri*) downregulate the expression of HPV E6 and E7 oncogenes [60,67]. *Lactobacillus* reduces the expression of matrix metalloproteinase-9 (MMP9), an enzyme that contributes to extracellular matrix degradation, cancer cell invasion, and metastasis [68]. By regulating key markers such as E-cadherin and β-catenin, it also helps inhibit the epithelia–mesenchymal transition, a critical process in cancer metastasis [69]. Research has demonstrated that the Wnt/β-catenin signaling pathway plays a critical role in the progression of CIN [70,71]. As CIN advances from low- to high-grade lesions, there is a notable increase in β-catenin expression and a shift in its localization from the cell membrane to the cytoplasm and nucleus [71]. This nuclear accumulation of β-catenin is a hallmark of active Wnt signaling. Once inside the nucleus, β-catenin acts as a transcriptional co-activator, promoting the expression of oncogenic target genes, such as the *c-Myc*, *Cyclin D1*, *Survivin*, *Sox2*, *Oct4*, and *Nanog* [71]. These genes support cellular proliferation, inhibit apoptosis, and enhance the stem-like characteristics of dysplastic cervical cells contributing to lesion persistence and progression toward cervical cancer.

Several studies have highlighted the influence of *Lactobacillus* strains on epithelial health and signaling pathways relevant to cancer and inflammation. For instance, *L. rhamnosus* GG (LGG) demonstrated protective effects in neonatal necrotizing enterocolitis by modulating intestinal stem cell activity, restoring tight junction integrity, and activating the Wnt/β-catenin signaling pathway [72]. Similarly, in colorectal cancer models, both *Lactobacillus* cocktails and *L. acidophilus* postbiotics were shown to interfere with proliferative signaling, including the Wnt, Notch, and BMP pathways, also exerting anti-proliferative and anti-migratory effects on cancer cells [73]. While these studies pertain to intestinal and colorectal models, they raise the question whether similar molecular interactions might be relevant in the context of CIN. Given the importance of the Wnt pathway in epithelial homeostasis and neoplastic progression, exploring the potential of specific *Lactobacillus* strains to influence CIN regression through similar mechanisms could be of interest. Some evidence also suggests that *Lactobacillus* strains might contribute to β-catenin regulation by modulating upstream components such as glycogen synthase kinase-3β, which facilitates β-catenin phosphorylation and subsequent degradation [74,75]. The exact relevance of this interaction in cervical tissue remains to be elucidated. However, it may offer a potential mechanistic explanation for the observed anti-proliferative effects of *Lactobacillus* in epithelial models.

Several studies have demonstrated that *Lactobacillus* could modulate host gene expression at the post-transcriptional level through the regulation of tumor-suppressor microRNAs (miRNAs), small non-coding RNAs that play a key role in cell cycle control, apoptosis, and oncogenesis [76,77,78]. In germ-free mice, microbial colonization altered the expression of intestinal miRNAs—miR-146, miR-375, and miR-10a, which regulate immune signaling pathways, including the Toll-like receptor (TLR)/MyD88 and cytokine balance (IL-12/IL-23) [79,80,81]. The dysregulation of these miRNAs contributes to inflammation and carcinogenesis. In colorectal cancer, certain lncRNAs (HOTAIR) were found to suppress tumor-suppressor miRNAs, promoting proliferation and therapy resistance [82]. Probiotic strains, including various *Lactobacillus* spp., were shown to modulate host miRNA profiles by decreasing oncogenic miRNAs (i.e., miR-21 and miR-182) and increasing tumor-suppressive ones (miR-125a-5p and miR-375), leading to enhanced apoptosis and reduced inflammation via targeting the Bcl-2, COX-2, and iNOS pathways [78,83]. Some *Lactobacillus*-derived components, including exopolysaccharides, induced pro-apoptotic gene expression and suppressed anti-apoptotic signals in cancer cells [84,85]. Although these findings were primarily demonstrated in colorectal cancer models, it is plausible that similar *Lactobacillus*–miRNA interactions could influence the cervical microenvironment. Hypothetically, *Lactobacillus*-induced modulation of miRNA expression may affect local immune tolerance, epithelial integrity, or viral persistence—mechanisms critical to HR-HPV clearance and CIN regression. For instance, the downregulation of pro-inflammatory or oncogenic miRNAs (miR-21) or the enhancement of miRNAs involved in epithelial differentiation and immune activation might support viral clearance and limit neoplastic progression. However, this remains speculative and warrants direct investigation in HPV-related cervical disease. Emerging evidence from vaginal microbiota studies supported a potential regulatory relationship between *Lactobacillus* dominance and host miRNA expression [86]. A study profiling miRNA expression in vaginal samples from young women revealed that non-*Lactobacillus*-dominated communities were associated with a broad upregulation of host miRNAs, including miR-23a-3p and miR-130a-3p. These miRNAs not only discriminated microbial community types with high accuracy, but they might also reflect or influence host immune or epithelial responses. Notably, *L. crispatus* dominance correlated with the most distinct miRNA profile, further supporting a functional link between beneficial microbes and local gene regulation. The *Lactobacillus*-associated suppression of specific miRNAs could contribute to a protective mucosal environment, potentially favoring HPV clearance or limiting progression to CIN. Although the causality remains to be established, this study offers a valuable molecular framework for exploring how vaginal commensals might shape host gene expression through miRNA modulation [86].

Modulating the immune response, through the activation of macrophages and dendritic cells, plays a crucial role in combating HPV infections [87]. Dendritic cells (DCs) are pivotal for initiating the host’s immune defense against HPV. Upon encountering the virus, DCs mature and produce type I IFNs and IL-12, essential for activating T cells and orchestrating an effective immune response [88]. However, HPV has developed mechanisms to evade this response, leading to persistent infections. Immunotherapeutic strategies aim to enhance DC function to counteract this evasion [89]. Macrophages also significantly contribute to the immune response against HPV. Studies show that HPV-positive head and neck squamous cell carcinomas contained a higher proportion of M1 macrophages, which are associated with anti-tumor activity, compared to HPV-negative tumors [90]. This suggests that modulating macrophage activity could influence disease outcomes. Macrophages play a key role in immune defense, including HPV infections. While some studies show a correlation between macrophage presence and HPV lesion grade, their exact role remains unclear [90]. Tumor-associated macrophages can suppress immune responses in cancer, and similar mechanisms may affect HPV infections [91].

HPV can interfere with innate immune mechanisms, such as downregulating TLR9 expression in infected cells, thereby evading immune detection [92]. Understanding these interactions has led to the exploration of therapies targeting TLR9 and other pathways to restore effective immune surveillance. Research demonstrated that cervical epithelial immune responses differed significantly between women with persistent HPV infections and those who cleared the virus [67]. The study revealed that type I IFNs and TLR3 were downregulated in persistent cases. Certain *Lactobacillus* strains, particularly *L. gasseri* LGV03 isolated from women who had cleared HPV, modulated immune signaling pathways. *L. gasseri* LGV03 enhanced antiviral signaling by increasing IRF3 phosphorylation in response to poly (I:C) stimulation and simultaneously attenuated the inflammatory response by downregulating NF-κB pathway activation. Additionally, *L. gasseri* LGV03 suppressed cervical cell proliferation in a zebrafish model, suggesting a potential role in supporting antiviral immunity and limiting pathogen-driven inflammation [67].

**Figure 2:**
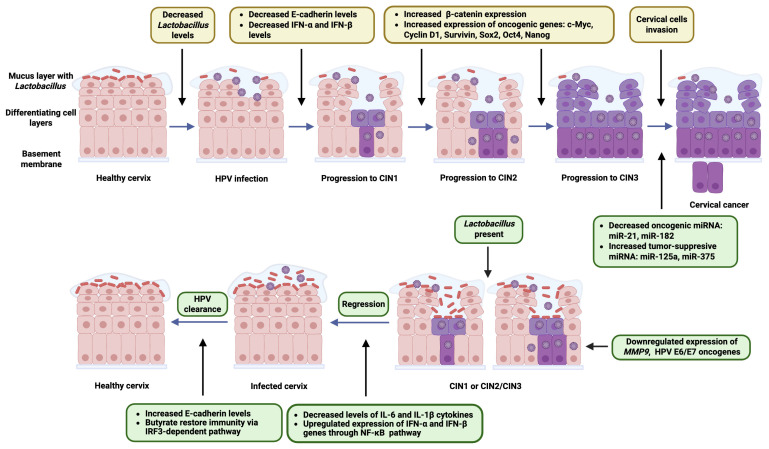
The figure illustrates the protective mechanisms by which *Lactobacillus* species act against HPV infection and cervical cancer progression. *Lactobacillus* enhances epithelial barrier integrity by increasing E-cadherin levels and promotes antiviral immunity by upregulating IFN-α and IFN-β through NF-κB signaling. It reduces chronic inflammation by lowering IL-6 and IL-1β levels and supports immune restoration via butyrate and IRF3 pathways. Certain strains suppress HPV oncogenes E6/E7 and inhibit *MMP9* expression, limiting invasion and metastasis. *Lactobacillus* also modulates Wnt/β-catenin signaling, preventing nuclear β-catenin accumulation and oncogene activation. Additionally, it regulates host miRNA expression—downregulating oncogenic miRNAs and upregulating tumor-suppressive ones—enhancing apoptosis and reducing inflammation.

Long-term HPV persistence leads to viral DNA integration into the host genome, disrupting key tumor suppressor genes and promoting carcinogenesis. Vaginal lactobacilli protect against HPV through mechanisms, such as epithelial barrier reinforcement, anti-inflammatory effects, and the modulation of immune responses [60]. HPV E6 expression was reduced by *L. crispatus* and *L. iners* and increased by *Gardnerella vaginalis*, *Megasphaera micronuciformis*, and *Fannyhessea vaginae* [93,94]. Similarly, E7 protein production was suppressed by *L. crispatus* and *L. gasseri* and enhanced by *L. iners*, *G. vaginalis*, and *M. micronuciformis* [60,95]. The latter species also reduced p53 and pRb tumor suppressor levels, potentially promoting cell cycle deregulation and neoplastic initiation [96]. Additionally, non-vaginal *Lactobacillus* strains showed promise as anti-cervical cancer probiotics. *L. casei* LH23 inhibited HeLa cell proliferation, reduced cell migration, and suppressed HPV E6 and E7 expression [97]. *L. casei* and *L. paracasei* also enhanced apoptosis-related gene expression while downregulating anti-apoptotic Bcl-2, suggesting a general anticancer effect [98,99]. Probiotic interventions, including vaginal *Lactobacillus* strains and non-vaginal *Lacticaseibacillus casei*, showed promise in reducing HPV infection and cervical cancer risk [60] (Figure 2).

## 5. Current Evidence Linking Vaginal Microbiota Composition to HR-HPV Persistence and CIN Outcomes

Several studies explored the association between the *Lactobacillus*-rich microbiota and the efficiency of HR-HPV elimination. The analysis of multiple studies revealed that the presence of *Lactobacillus* species in the cervicovaginal environment was associated with a decreased detection of HR-HPV infections, CIN, and cervical cancer. Specifically, *L. crispatus* was identified as a critical protective factor, correlating with lower rates of HR-HPV and CIN [100,101]. In a cohort of 73 women analyzed, 58.9% experienced HPV clearance within a 12-month period [102]. There were no statistically significant differences between those who cleared the virus and those who did not in terms of the age, stage of disease, HPV subtype, vaginal microbiota CSTs, or vaginal microbiota diversity (both α and β metrics). At baseline, participants with a lower presence of *Enterococcus* ASV 62 and higher levels of *L. iners* were less likely to eliminate the virus by the 12-month mark. Additional analysis indicated a notable inverse correlation between elevated *L. iners* abundance and HPV clearance in individuals undergoing non-surgical treatment, whereas no such association was found among those who received surgical interventions [102].

A pilot study by Di Paola et al. [6] evaluated HPV testing in a primary screening program involving 1029 women aged 26–64. Multiple HPV genotypes were detected in 13 cases. After one year, all HR-HPV-positive women underwent follow-up testing and were classified into the clearance group (*n* = 27) (i.e., women who had cleared the infection (no HR-HPV DNA detected)) and the persistence group (*n* = 28), i.e., women with a persistent HR-HPV infection (at least one HPV genotype remained). *Lactobacillus* was the dominant genus across all groups (clearance, persistence, and control), but differences in species distribution were observed. *L. crispatus* was the most abundant species in both the control and clearance groups, suggesting a potential role in HPV clearance [6] (Table 1).

Zeng et al. [103] investigated the relationship between vaginal microbiota composition and the clearance of HR-HPV infection. Among 135 participants, including 45 HPV-negative and 90 patients with an HPV infection, 26 patients experienced HPV clearance within a year, with most turning negative within six months. The analysis revealed that women with HPV clearance had significantly lower bacterial alpha diversity. The lower diversity of the vaginal microbiota, characterized by a predominance of *Lactobacillus* species, may be associated with natural HPV clearance. In contrast, persistent HPV infection could be linked to the presence of specific bacterial taxa, including *Fusobacterium*, *Neisseria*, *Helicobacter*, and Bacteroides (genus level), as well as *Streptococcaceae*, *Neisseriaceae*, *Helicobacteraceae*, *Erysipelotrichaceae*, and *Bacteroidaceae* (family level) [103]. Brotman et al. [104] explored the relationship between vaginal microbiota compositions and the detection of HPV. Thirty-two women of reproductive age self-collected mid-vaginal swabs twice a week over a 16-week period, yielding a total of 937 samples. By sequencing barcoded 16S rRNA genes, the study identified six distinct CSTs within the vaginal bacterial communities. Each swab was also tested for the presence of 37 different HPV types. The study revealed that HPV clearance was strongly associated with the structure of the vaginal microbiota. Microbial communities dominated by *L. gasseri* were linked to a faster clearance of the virus, whereas those characterized by low *Lactobacillus* abundance and a higher presence of *Atopobium* were associated with a delayed clearance. Additionally, certain low-*Lactobacillus* community types appeared to increase the likelihood of acquiring new HPV infections [104].

**Table 1:** Overview of the research on cervicovaginal microbiota composition and its impact on HPV infection dynamics and cervical neoplasia.

Author, Year; Country [Ref]	Study Aim	Groups: Cases and HPV-Negative Controls	Material and Detection Method	Results	Changes in Microbiota Abundance	Conclusions
Brotman et al., 2014; USA [104]	Assess temporal links between microbiota dynamics and HPV detection	32 women, 937 self-collected vaginal samples over 16 weeks	Vaginal swabs; 16S rRNA sequencing; HPV DNA testing	CST II (*L. gasseri*) linked to HPV clearance; CST IV-B (anaerobes) linked to persistence; the most abundant *Lactobacillus* species in the control and clearance group	*L. gasseri* enriched in clearance; *Atopobium* in slow clearance states	Microbiota state changes HPV detection and outcomes; longitudinal sampling is key
Di Paola et al., 2017; Italy [6]	Characterize cervicovaginal microbiota in persistent HR-HPV	55 HPV-positive (from 1029 screened), 17 HPV-negative controls	Cervicovaginal swabs; 16S rRNA pyrosequencing	CST IV more frequent in persistent HPV (72.7%) vs. controls (16.6%)	Decreased *Lactobacillus*, increased *Gardnerella*, and *Atopobium* in the persistent group	*Lactobacillus*-dominated microbiota linked to HPV clearance; dysbiosis favors persistence
Mitra et al., 2020; UK [31]	Study how vaginal microbiota influences CIN2 lesion regression	87 women (16–26 years) with CIN2, followed-up for 24 months	Vaginal swabs; 16S rRNA sequencing	*Lactobacillus*-dominant microbiota associated with lesion regression	Increased *Gardnerella, Prevotella*, and *Megasphaera* in the persistent group	Vaginal microbiota could predict CIN2 outcome and serve as a therapeutic target
Zeng et al., 2023; China [103]	Explore vaginal microbiota’s role in HPV infection, persistence, and clearance	90 HPV-positive (persistent vs. cleared), 45 HPV-negative controls	Vaginal swabs; 16S rRNA sequencing	Higher alpha diversity in persistent HPV; *L. iners* prevalent in infections	Increased *Sneathia amnii*, *Bacteroidaceae* in persistence; *L. crispatus* in clearance	Microbiota composition affects HPV outcomes; *L. crispatus* dominance may promote clearance

CIN, cervical intraepithelial neoplasia; CSTs, community state types, for classification of distinct vaginal microbial communities.

A study conducted by Mitra et al. [31] provided detailed scientific data on the association between vaginal microbiota composition and the regression of CIN2. The study included 87 participants. They were 16–26 years old, with histologically confirmed CIN2. The follow-up period was 24 months. At baseline, CST III (*L. iners*-dominant; 41.4%) was the most common, followed by CST IV (*Lactobacillus*-depleted; 34.5%) and CST I (*L. crispatus*-dominant; 24.1%). CST II (*L. gasseri*) and CST V (*L. jensenii*) were absent at baseline but occurred in several samples later on. Vaginal microbiota composition did not significantly differ based on HPV status or subtype at baseline. *Lactobacillus* depletion and the presence of specific anaerobic bacteria at CIN2 diagnosis were linked to a significantly lower likelihood of regression at 12 and 24 months. When regression occurred, it was slower in the absence of a *Lactobacillus*-dominant vaginal microbiota. A similar trend was observed at 12 months in women with persistent disease, though the statistical power is limited due to the reduced sample size. The evidence suggests that interventions aimed at restoring and maintaining a healthy vaginal microbiota, including probiotic therapies, could be beneficial in HPV infection management and cervical dysplasia treatment.

Liu et al. [105] showed the effects of the intravaginal administration of *L. crispatus* chen-01 on HR-HPV infections. In this study, 100 women with HR-HPV were randomly assigned to receive either the probiotic treatment or a placebo. After six months, the group treated with *L. crispatus* chen-01 showed a significant reduction in HPV viral load, an increased clearance rate of the virus, and improved vaginal health without notable adverse effects. Additionally, 16S rRNA sequencing indicated that the probiotic effectively restored a healthy vaginal microbiota composition in these women. These findings suggest that *L. crispatus* chen-01 could be a promising treatment option for HR-HPV infections [105].

## 6. Therapeutic Perspectives

The modulation of the vaginal microbiota using probiotics and prebiotics, particularly those containing immunomodulatory and acid-producing *Lactobacillus* species, has emerged as a promising adjunctive strategy in the management of HR-HPV infections. Several clinical studies investigated the therapeutic potential of probiotics in the context of HR-HPV infections and cervical dysplasia, employing diverse formulations, strains, and routes of administration (Table 2). Interventions included oral and vaginal deliveries of specific *Lactobacillus* strains administered either as a monotherapy or as adjuncts to the standard treatment. Probiotics may offer several therapeutic benefits when used alongside conventional treatments for CIN, such as the loop electrosurgical excision procedure [106,107]. These benefits include promoting tissue repair, reducing post-treatment inflammation, restoring healthy microbial balance, and potentially decreasing the risk of HR-HPV reinfection or disease progression. Moreover, microbiota restoration may also contribute to a reduction in dysbiosis-associated symptoms such as vaginal discomfort, discharge, or an increased susceptibility to secondary infections [61]. The integration of probiotics into treatment regimens may be particularly valuable in patients with persistent HPV infections or incomplete immune clearance following the standard therapy. Probiotic formulations administered orally or intravaginally are being evaluated in ongoing trials to assess their role in accelerating viral clearance and reducing treatment-related complications.

**Table 2:** Summary of clinical studies on probiotic interventions in HR-HPV clearance and cytological outcomes.

Number of Clinical Trial	Study Period, Time Frame (Months); and Location	Intervention/Treatment	Inclusion Criteria	Exclusion Criteria	Study Design and Masking	Sample Size	Study Group	Control Group	Primary Outcome Measures	Secondary Outcome Measures
NCT01097356	2010–2011 (6); Belgium	Probiotic drink vs. no intervention	Women; age 18–65; LSIL+ HPV+ in the Papanicolaou test	Age >65; immuno-compromised	Randomized, parallel; single-blind (participant)	60	Probiotic drink daily (6 months)	No intervention (6-months observation)	HPV positivity; LSIL regression	No data
NCT01599416	2011–2013 (12); Taiwan	Oral U-relax (*L. rhamnosus* GR-1 + *L. reuteri* RC-14) vs. placebo	Women; age 30–65; HPV+ 6 months after conization; negative for intraepithelial lesion or malignancy in the Papanicolaou test	CIN before conization, cervical cancer, genital infection issues, long-term antibiotics	Randomized, parallel; triple-blind (participant, care provider, investigator)	80	U-relax: 2 caps/day → 1/day until day 360	Placebo (identical schedule)	Vaginal health status	HPV DNA index change
NCT03372395	2015–2016 (9); Italy	Vaginal *L. rhamnosus* BMX 54 + standard treatment	Age >18, bacterial vaginosis/yeast vaginitis + HPV; (ASCUS, LSIL, CIN1, HPV DNA+)	Pregnancy; CIN2/3, cancer, immuno-deficiency, corticosteroids	Randomized, sequential (pilot); open label	117 (60 short, 57 long)	Long-term probiotics (6 months)	Short-term probiotics (3 months)	Infection symptom resolution	Adverse events (CTCAE v4.0)
NCT05109533	2018–2021 (12); Italy	Vaginal + oral probiotics: *L. rhamnosus* BMX 54, *L. reuteri* RC-14, *L. rhamnosus* GR-1 + standard treatment	Age >18, positive vaginal infection swabs, HPV+	Pregnancy or breast-feeding, malignancies, immunological diseases, comorbidities, corticosteroids	Randomized, parallel; open label	483 (252 probiotic, 231 controls)	Standard treatment + 9-months probiotics	Standard treatment	HPV clearance; vaginal infection resolution	No data
NCT06802809	2022–2024 (4); Italy	Oral *L. crispatus* M247 (Crispact^®^) vs. placebo	Women; age 18–69, HR-HPV+, ASCUS or LSIL; negative colposcopy (biopsy)	Prior HPV vaccination, HSIL, immunotherapy, neoplasia, pregnancy, allergy	Randomized, parallel; placebo-controlled, single-blind (participant)	66	*L. crispatus* M247, 20 billion CFU/day for 4 months	Placebo, 1 stick/day for 4 months	HR-HPV clearance	Microbiota change, cytology normalization, side effects

ASCUS, atypical squamous cells of undetermined significance; CTCAE, Common Terminology Criteria for Adverse Events; CIN, cervical intraepithelial neoplasia; HSIL, high-grade squamous intraepithelial lesion; LSIL, low-grade squamous intraepithelial lesion.

Dellino et al. [108] noted that the oral administration of *L. crispatus* M247 was associated with a higher probability of resolving HPV-related cytological abnormalities compared to those who only underwent follow-up (60.5% vs. 41.3%). The median follow-up was 12 months (ranging from 10 to 30 months). Complete HPV clearance was observed in 9.3% of the patients in the follow-up-only group, whereas 15.3% of those taking long-term oral *L. crispatus* M247 achieved clearance. However, by the end of the study, the proportion of HPV-negative patients, as determined with the HPV-DNA test, was not significantly different between those two groups [108].

A randomized study included 117 women divided into two groups, as follows: one received standard antimicrobial treatment plus a short-term (3-month) course of vaginal probiotics, while the other received the same treatment followed by a long-term (6-month) course of *L. rhamnosus* BMX 54 [109]. The results showed that the long-term probiotic group experienced a higher rate of HPV clearance (31.2%) compared to the short-term group (11.6%). This suggests that extended use of vaginal *L. rhamnosus* BMX 54 may help restore a balanced vaginal microbiota and aid in resolving HPV infections. Another study included 121 women with a genital HR-HPV infection, divided into a study group (62 participants) and a control group (59 participants) [110]. There was no significant difference in the HR-HPV clearance rates between the groups (58.1% vs 54.2%). A lower initial viral load was the only factor predicting HR-HPV clearance. Twenty-two women had a mildly abnormal initial cervical smear and nine had an unsatisfactory smear. At a 6-month follow-up, both mildly abnormal cervical smear and unsatisfactory smear rates decreased significantly in the study group compared to the control group. The application of probiotic strains (i.e., *L. rhamnosus* GR-1 and *L. reuteri* RC-14) did not influence genital HR-HPV clearance but may have decreased the rates of mildly abnormal and unsatisfactory cervical smears.

In parallel, the impact of prophylactic HPV vaccination on the composition and function of the vaginal microbiota is gaining increasing scientific attention [111,112]. Although prophylactic HPV vaccines primarily target viral infection and lesion development, emerging evidence suggests that vaccination may also influence the vaginal microenvironment. By reducing chronic-HPV-related inflammation and epithelial disruption, vaccination could indirectly support the restoration or maintenance of a healthy *Lactobacillus*-dominated microbiota [43]. Furthermore, the interplay between host immunity and microbial composition can be modulated by vaccine-induced immune responses, which affect microbial dynamics and resilience against dysbiosis [113]. While vaccines targeting oncogenic HPV types have proven highly effective at reducing infection rates and associated lesions, their influence on the local microbial ecosystem remains incompletely understood. *Lactobacillus*-based strategies may serve as complementary tools to vaccination by enhancing mucosal resilience, supporting a balanced immune response, and preventing chronic, subclinical infections. Combining prophylactic vaccination with microbiota-supportive interventions could offer a synergistic approach to preventing both primary HPV infections and their recurrence in at-risk populations. Future research should aim to clarify the interplay between vaccination, host immunity, and microbial dynamics to optimize long-term protective strategies against HPV-related disease.

## 7. Conclusions

The effective clearance of HR-HPV is essential for interrupting the pathway to cervical carcinogenesis, and the modulation of the vaginal microbiota through *Lactobacillus*-dominated communities may play a crucial role in achieving this outcome. Key findings from studies on the interactions between the vaginal microbiota and HR-HPV infections highlight the pivotal role of specific *Lactobacillus* species in facilitating viral clearance and supporting the regression of cervical dysplastic lesions. Species such as *L. crispatus* and *L. gasseri* exhibit protective effects through the modulation of local immune responses, the maintenance of acidic vaginal pH, and the competitive inhibition of pathogenic microorganisms. Given the current evidence, the use of probiotics containing selected *Lactobacillus* strains emerges as a promising adjunctive strategy in the management and prevention of HPV-related cervical abnormalities. However, further research, including randomized clinical trials, is essential to validate their efficacy, safety, and to establish optimal treatment protocols. Moreover, the individualization of probiotic-based interventions, tailored to the specific microbial profile of each patient, may significantly enhance therapeutic outcomes. Integrating microbiological, gynecological, and immunological insights opens new avenues for the development of innovative, microbiota-targeted strategies in the fight against HR-HPV and cervical dysplasia.

## Data Availability

Not applicable.

## Abbreviations

The following abbreviations are used in this manuscript:

HR	High risk
HPV	Human papillomavirus
CIN	Cervical intraepithelial neoplasia
LR	Low risk
pRB	Retinoblastoma protein
CST	Community state type
BV	Bacterial vaginosis
AV	Aerobic vaginitis
IFN	Interferon
IL	Interleukin
TLR	Toll-like receptor
IRF	Interferon regulatory factor
miRNA	microRNA
DC	Dendritic cell
